# Waldo: Automated discovery of adverse events from unstructured self reports

**DOI:** 10.1371/journal.pdig.0001011

**Published:** 2025-09-30

**Authors:** Karan S. Desai, Vijay M. Tiyyala, Pranav Tiyyala, Atharva Yeola, Alejandra Gallegos-Rangel, Alejandro Montiel-Torres, Matthew R. Allen, Mark Dredze, Ryan G. Vandrey, Johannes Thrul, Eric C. Leas, Mike Hogarth, Davey M. Smith, John W. Ayers

**Affiliations:** 1 University of Michigan Medical School, University of Michigan, Ann Arbor, Michigan, United States of America; 2 Department of Computer Science, Johns Hopkins University, Baltimore, Maryland, United States of America; 3 All India Institute of Medical Sciences, Bhubaneswar, India; 4 Department of Electrical & Computer Engineering, University of California San Diego, La Jolla, California, United States of America; 5 The Qualcomm Institute, University of California San Diego, La Jolla, California, United States of America; 6 ENLACE Summer Research Program, University of California San Diego, La Jolla, California, United States of America; 7 School of Medicine, University of California San Diego, La Jolla, California, United States of America; 8 Altman Clinical Translational Research Institute, University of California San Diego, La Jolla, California, United States of America; 9 Department of Psychiatry and Behavioral Sciences, School of Medicine, Johns Hopkins University, Baltimore, Maryland, United States of America; 10 Department of Mental Health, School of Public Health, Johns Hopkins University, Baltimore, Maryland, United States of America; 11 Herbert Wertheim School of Public Health and Human Longevity Science, University of California San Diego, La Jolla, California, United States of America; 12 Division of Biomedical Informatics, Department of Medicine, University of California San Diego, La Jolla, California, United States of America; 13 Division of Infectious Diseases and Global Public Health, Department of Medicine, University of California San Diego, La Jolla, California, United States of America; Shiraz University of Medical Sciences, IRAN, ISLAMIC REPUBLIC OF

## Abstract

Adverse event (AE) detection is labor-intensive and costly given the task is to find rare events. Automated solutions to enhance efficiency, reduce costs, and capture unnoticed safety signals are needed. To develop and evaluate an automated machine learning tool, “Waldo,” for AE detection from unstructured social media text data, specifically targeting consumer health products that lack traditional post-market surveillance channels. We tested three models – (i) N-gram model, (ii) BERT (Bidirectional Encoder Representations from Transformers), and (iii) RoBERTa (Robustly optimized BERT approach) – trained on 10,000 previously published unstructured reports on cannabis-derived products (CDPs) annotated by humans for the presence of adverse events to determine the best-performing AE detection method. This method was then benchmarked against an AI chatbot (ChatGPT: gpt-3.5-turbo-0613) and applied to previously unstudied user narratives about CDPs from 20 subreddits.RoBERTa demonstrated the highest accuracy at 99.7%, hereafter referred to as Waldo, with 22 false positives and 12 false negatives, yielding an F1-score of 95.1% for the positive class. In contrast, the chatbot had an accuracy of 94.4%, with 401 false positives (18.23-fold more than Waldo) and 163 false negatives (13.58-fold more than Waldo), yielding an F1-score of 38% for the positive class. Applying Waldo to 437,132 posts identified 28,832 potential AEs. The subreddit r/Marijuana had the highest AE rate (12.7%), followed by r/weed (10.5%) and r/AskTrees (10.0%). r/weedstocks (0.1%), r/macrogrowery (0.2%), and r/weedbiz (0.2%) had the lowest rates of potential AEs. Waldo addresses critical gaps in safety surveillance for unregulated consumer health products by automatically detecting adverse events from social media—a capability absent in traditional industry systems. Unlike existing approaches limited to structured databases or narrow domains, Waldo processes informal user narratives at scale with high precision. We have open-sourced Waldo for immediate application by the health community [https://waldo-ae-detection.github.io/WALDO/].

## Author summary

Adverse events—unexpected health problems linked to products—are usually reported through official systems that are slow, limited, and difficult for the public to use. At the same time, millions of people share their experiences openly on social media, including negative health effects from products like cannabis. Until now, most of this information has gone unnoticed by health agencies. We created **Waldo**, a machine learning tool that can automatically scan large amounts of unstructured text, such as Reddit posts, to identify when people describe possible AEs. Waldo performed with very high accuracy and allowed us to quickly find tens of thousands of potential health concerns in online discussions. We also confirmed that most of the posts Waldo flagged truly reflected adverse events. By making Waldo open-source and freely available, we aim to provide researchers, clinicians, and public health officials with a scalable way to detect safety signals earlier, more efficiently, and in communities that are often overlooked.

## Introduction

Post-market safety surveillance systems, such as the FDA Adverse Event Reporting System (FAERS) or MedWatch, are crucial for public health and safety but neglect a wide range of consumer products, including wellness or recreational items like cannabis-derived products. This neglect is exacerbated because approved prescription medications and medical devices heavily rely on industry sentinels for safety signals which do not exist for wellness or recreational products. Most adverse event (AE) reports received by regulatory agencies originate from third-party gatekeepers such as the biopharmaceutical companies, lawyers, and clinicians. For instance, up to 98% of device AE reports on medical devices originate from the device manufacturers themselves [1,2]. The narrow scope of AE reporting channels, inherent bureaucracy, and potential conflicts of interest lead to significant information and time gaps.

Although not originally designed to handle the safety signals emerging from the rapid growth in consumer health products [3,4], regulatory bodies have increasingly prioritized the development of direct feedback channels [5]. In 2007, the U.S. Food and Drug Administration (FDA) introduced the MedWatch Consumer Voluntary Reporting form (3500B) to enable consumers to report potential safety concerns directly to regulators. However this safety reporting system remains largely unknown to the general public and is cumbersome to navigate. For example, if a user attempts to report an adverse event related to cannabidiol (CBD) and selects the most reasonable option “other products not listed” from the initial dropdown menu, they will not be prompted to complete the MedWatch. Consequently, AEs related to cannabis-derived products are under-reported to the FDA relative to their occurrence in the wild [6]. This is of significant public health importance due to the broad use of cannabis products in the U.S. and the variability in production quality and safety practices [7]. As demonstrated by the lung injuries from vaping cannabis products [8], these variations can result in substantial harm.

Nevertheless, the public continually voices concerns about health-related products, particularly widely used commercial items, through social media and other informal channels rather than through standard reporting systems [1,9]. This disconnect underscores significant gaps in safety surveillance systems. Extracting spontaneous AE reports from unstructured data presents significant challenges. The rarity of these events makes their identification akin to finding a needle in a haystack, rendering AE detection not only labor-intensive but also prone to false positives. Current methods predominantly rely on manual review, which is time-consuming and susceptible to human error [10]. For example, a large staff whose primary task was reviewing potential safety reports required a median of 1 hour and 9 minutes of reading/reviewing to detect each valid AE signal [11].

Automated solutions that streamline this process and improve the detection of critical safety signals are vital in the era of data-driven healthcare [9]. However, most work in this space has been commercial, often relying on “black-box” systems that lack transparency in their mechanisms and accuracy, leaving regulators in the dark and limiting adoption. Meanwhile, academic research typically focuses on one-off studies aimed at finding substantive results for a specific product, with less emphasis on developing tools to equip the broader community with AE detection capabilities. For example, public tools like the ones available on GitHub (e.g., https://github.com/andreped/adverse-events) may focus on narrow subject areas and primarily provide codebases for replication rather than advancing the research.

We aim to fill this reporting void by developing an AI-powered tool named “Waldo” to automatically detect AEs from unstructured text data. Here, we provide metrics regarding the development and evaluation of Waldo, using a case series of consumer reports on cannabis-derived products - selected due to their potentially overlooked safety concerns related to poor quality control in product manufacturing and labeling [12–14]. These concerns are exacerbated by minimal proactive FDA surveillance or safety standards, as cannabis-derived products are mostly unregulated at the federal level [15,16]. Unlike drugs, devices, and biologics, which undergo rigorous pre-market testing and require proof of efficacy and safety, cannabis products do not face the same scrutiny. Furthermore, this industry has a history of overstating the benefits while minimizing potential harms of their products [17]. To promote widespread adoption and enhance safety practices, we made Waldo open-source, thereby, democratizing access to advanced AE detection technology.

## Methods

Our analysis strategy was two-phased. In Phase 1, we report on the strategies used to identify the method that achieved the most accurate predictions compared to ground-truth human annotations. In Phase 2, we show how Waldo, the most accurate model from phase 1, can be used for AE detection in practice by performing a demonstrative analysis of user-authored reports about CDPs.

### Waldo development

The training data and annotations to inform the development of Waldo were sourced from a previously published peer-reviewed study, where human annotators identified AEs [6]. This dataset was obtained from Reddit’s r/Delta8 posts (N = 65,200) from April 14, 2020 (the inception of r/Delta8), through September 25, 2022. To identify AEs among delta-8-THC users, the team randomly sampled 10,000 original posts, with a mean (SD) of 39 (84) words, for further annotation. Human annotators, using double annotations and resolving disagreements in collaboration with the study PI, identified 335 potential AE reports [5]. We selected these data because they represent an entire population (all r/Delta8 posts), were thoroughly adjudicated, and pertains to a product that may be overlooked by FDA sentinels.

To replicate the human evaluators’ AE annotations, we considered three classification models: (i) N-gram model, which uses traditional bag-of-words and frequency-based approaches to represent text as a combination of adjacent word sequences; (ii) BERT [18], a transformer-based model that captures contextual information bidirectionally and has been fine-tuned for text classification tasks; and (iii) RoBERTa [19], an optimized version of BERT that improves on the pretraining process by using larger mini-batches and a longer training duration, leading to better contextual understanding and classification accuracy. Hyperparameter optimization was conducted by systematically evaluating learning rates [1e-5, 2e-5, 3e-5, 5e-5] and batch sizes [8,16,32], with models trained for up to 50 epochs using early stopping (patience = 9 epochs). The optimal configuration selected based on validation AUC-ROC performance used learning rate = 2e-5 and batch size = 16. Training employed AdamW optimizer with gradient clipping and linear learning rate scheduling. The performance of these classifiers was assessed using accuracy, precision, recall, F1-score, and confusion matrices.

### Waldo Comparison with a Large Language Model (LLM) Chatbot

We compared the accuracy of Waldo against a chatbot using the same training data [6]. ChatGPT (gpt-3.5-turbo-0613) was set to the default settings (Temperature = 1, Top P = 1, Max token limit = 1700, Frequency Penalty = 0, and Presence Penalty = 0); given each Reddit post; and asked to reference annotation instructions identical to those given to human annotators that formed the basis of our training data **(Supplement A in** S1 File). The performance of the chatbot was assessed using the same strategies as applied to Waldo, including accuracy, precision, recall, F1-score, and confusion matrices, and the absolute differences in these performance metrics were described.

### Waldo demonstration

We obtained all posts from 20 relevant Reddit subreddits from their inception through March 31, 2022. These included ArtOfRolling (3982 posts), AskTrees (299 posts), bongs (2493 posts), Cannabis_Culture (2580 posts), CannabisExtracts (11825 posts), cannabis (328 posts), CBD (24678 posts), macrogrowery (2498 posts), Marijuana (14206 posts), microgrowery (36272 posts), MMJ (3042 posts), outdoorgrowing (7748 posts), rosin (8900 posts), StonerEngineering (5755 posts), stoner (3156 posts), trees (176799 posts), treedibles (21716 posts), weed (99545 posts), weedbiz (2888 posts), and weedstocks (8422 posts). We chose these subreddits due to their relevance to cannabis. We then applied the best performing model–hereafter known as Waldo–to these datasets to analyze the frequency of potential AE reports by subreddit. The results include both overall and subreddit-specific frequencies of potential AEs.

All analyses were conducted using Python. The UC San Diego and Johns Hopkins University IRB exempted the analyses from review since the study used public, non-identifiable data (45 CFR §46).

In summary, we first evaluated the three classification models on our annotated dataset to identify the best-performing approach, then compared this model against ChatGPT, and finally applied it to the broader Reddit dataset for real-world demonstration.

## Results

**Fig 1:**
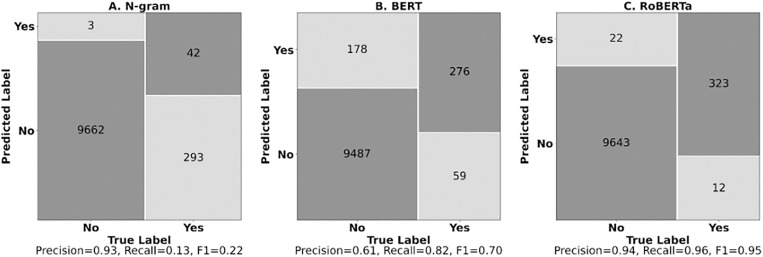
Confusion Matrices for Three Text Classification Models. Comparison of confusion matrices for N-gram (A), BERT (B), and RoBERTa (C) classifiers. Each matrix displays true positives, false positives, false negatives, and true negatives. RoBERTa achieved the highest accuracy (99.7%) and F1 score (0.95) for the positive class.

Following our three-phase evaluation strategy, we first assessed how well each classification model replicated human AE annotations. The classification models achieved varying levels of accuracy with increased model complexity. Classifier A (N-gram Model) correctly predicted 97.0% of outcomes, with 3 false positives and 293 false negatives (F1 = 0.22 for the positive class) (Fig 1). Classifier B (BERT), with an accuracy of 97.6%, had 178 false positives and 59 false negatives (F1 = 0.70 for the positive class). Classifier C (RoBERTa) delivered the highest accuracy at 99.7%, with 22 false positives and 12 false negatives (F1 = 0.95 for the positive class). Furthermore, Classifier C had a precision of 93.6% (the proportion of true positive predictions among all positive predictions) and a recall of 96.4% (the proportion of true positive predictions among all actual positive instances).

Qualitative review of RoBERTa’s 34 misclassifications revealed distinct patterns. False negatives (n = 12) primarily missed mental health (anxiety, paranoia) and physical symptoms (tongue/lung irritation), while false positives (n = 22) occurred when symptoms were discussed as secondhand reports (what users had heard from friends or online) or in positive contexts (e.g., “body not aching after using delta-8”).

The AI-powered chatbot had an accuracy of 94.4%, with 401 false positives and 163 false negatives (F1 = 0.38 for the positive class). Alternatively framed, in contrast to Waldo the chatbot had 18.2 times more false positives and 13.6 times more false negatives. Moreover, the chatbot had a precision of 30.0% and a recall of 51.3%, also less than Waldo. Additional results are available in the **Supplement B in** S1 File.

**Fig 2:**
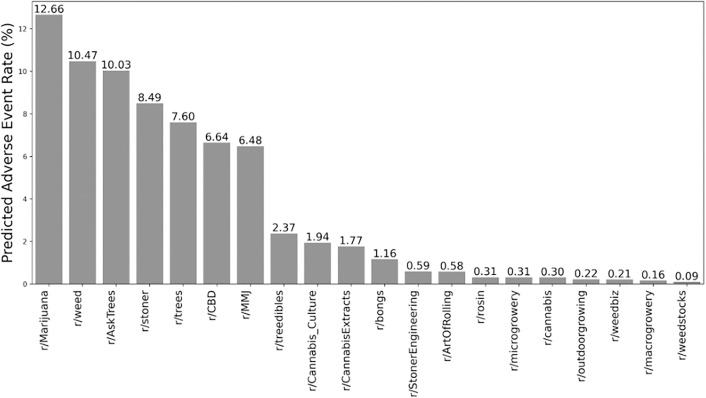
Adverse Event Rates Across Cannabis-Related Subreddits. Percentage of posts containing potential adverse events (AEs) in various cannabis-related subreddits. r/Marijuana showed the highest AE rate (12.7%), while r/weedstocks had the lowest (0.1%).

Applying the best performing model, Classifier C–hereafter referred to as Waldo–to 437,132 posts from 20 subreddits, we identified 28,832 potential AEs. From this collection of 28,832 potential AEs, we randomly selected 250 for validation, of which 215 (86.0%) were confirmed to be AEs by double annotator review. **Fig 2** illustrates the rate of AEs by subreddit, highlighting how the tool can pinpoint potential AE data sources for further review.

The subreddit r/Marijuana had the highest rate of potential AEs (12.7%; 95%CI:12.11-13.21), followed by r/weed (10.5%; 95%CI:10.28-10.67), r/AskTrees (10.0%; 95% CI:6.69-13.71), and r/stoner (8.5%; 95%CI:7.54-9.47). These findings suggest that these subreddits may be particularly valuable sources for identifying potential adverse events associated with cannabis use.

**Table 1** includes examples of flagged posts, highlighting the diversity of content Waldo identifies as AE-relevant. One user shared how smoking a small amount of cannabis with low tolerance triggered severe panic attacks that lasted for days, while another reported experiencing tinnitus after CBD use, wondering if it was related. These examples highlight the diverse range of adverse events, from intense psychological reactions to unexpected physical symptoms, emphasizing the need for awareness of cannabis’ varying effects on individuals.

**Table 1:** Example reddit posts of flagged AEs for CDPs.

Reddit	Post
r/weed	I tried smoking weed my second time (first time was around 2/3 years ago and I didn’t feel anything) and it was awful… I ended up shaking because I didn’t eat anything and I felt the blood pressure drop and it all ended up with me puking… It’s now been around 5 weeks and I’ve kind of lost myself where I’m not able to feel as much as before. Meaning that I don’t feel the enjoyment and happiness and I feel like I’m unattached from my body. I’ve contacted the doctor and psychiatrist so I’m getting help. The thing is that I have other symptoms that I’m curious if anyone knows what they are. These are muscle twitching, insomnia (gotten a little better now), vivid dreams, derealisation from time to time, small panic attacks, small trouble concentrating, small problems with short term memory (this is something I’ve had before trying weed), feeling like my thoughts and my body are external from each other, eating more than I could before and a few others. It’s also been a thing with me thinking that I am developing schizophrenia. This is because I looked up my symptoms on forums days after the experience and it was mentioned that it could be a possibility. And that has been stuck in my head.
r/AskTrees	Help me please. I rarely smoke and I smoked maybe a bowl by myself and I have really low tolerance. I’ve never gotten panic attacks but I’ve been having panic attacks since 9pm Monday and it’s now 12:03 am on Wednesday… And I can’t calm down without laying on the ground and freaking out for a good 20 minutes… Should I get medical help
r/AskTrees	I took a massive dab last night, and with each passing minute it felt like I was just getting higher and higher. I eventually reached a point where it felt like I was almost tripping on acid, like if I relaxed for long enough that my ego would just completely disappear. It kinda felt like I broke through reality. I wasn’t really scared at first, but I knew I wanted to come down. When it felt like nothing had changed for hours, I started to worry if I was experiencing psychosis. Somehow, I was able to get home and wake up in my bed this morning. Has anybody else ever experienced something like this, and if so, what did you do that helped bring you down?
r/trees	…Whenever I smoke high THC cannabis I have “green outs” I pretty much have a panic attack and I feel like I’m gonna barf (my anxiety is based around my vomiting phobia). I can only smoke low THC high CBD flower now but I miss THC
r/CBD	Has anyone had tinnitus pop up from using CBD stop using it for a bit and taken a break and had the tinnitus go away? Have some ringing in my ears for several days which I think came from my cbd use, curious if anyone had similar experience and had any success with the ringing subsiding.

The lowest rates of AEs were found in r/weedstocks (0.1%; 95%CI:0.04-0.17), r/macrogrowery (0.2%; 95%CI: 0.04-0.32), and r/weedbiz (0.2%; 95%CI:0.07-0.38), places that a priori due to their content focus, such as investing or business development, were unlikely to solicit AE reports.

AE rates by subreddit were computed with 95% confidence intervals using bootstrap resampling (n = 10,000 iterations) with replacement in Python, ver 3.

## Discussion

Waldo demonstrated high accuracy (99.7%) and balanced performance in precision (96.4%) and recall (93.6%), eclipsing the capabilities of an AI-powered chatbot. By applying this model to CDP-related Reddit posts, we highlighted the effectiveness of automated methods in enhancing pharmacovigilance efforts and addressing underreported safety signals in consumer health products.

Detecting AEs remains crucial to safeguarding public health, especially as the market for consumer health products expands. The FDA has recognized the need for robust post-market surveillance systems, as demonstrated by the expansion of the Sentinel Initiative in 2019 [20]. This initiative was designed to integrate big data and advanced data science methods, allowing for more comprehensive and timely detection of safety signals. While the initiative effectively incorporates data from electronic health records (EHRs), hospital stays, and pharmaceutical dispensing, it lacks in capturing AEs informally reported in the wild. This gap is significant because online data have emerged as a prominent venue for users to share their health experiences, often more candidly than in medical settings [21].

Prior work has leveraged machine learning models to analyze unstructured text data, such as clinical notes, Amazon reviews, and social media posts, demonstrating the capability of these models to extract safety signals [22]. For example, Zhao et al. [23] have shown that machine learning can effectively analyze large-scale unstructured data for AE detection. Researchers have applied these techniques to specific areas, analyzing e-cigarette safety issues on forums [24] and mining diabetes drug reviews from patient-based health platforms like WebMD [25].

However, existing approaches to AE detection vary significantly in their sophistication and scope. Traditional methods, exemplified by the adverse-events tool [26], rely on SVMs and Random Forest algorithms applied to structured FAERS data. While broadly applicable, these approaches have limited natural language processing capabilities and cannot capture the nuanced, informal language characteristic of social media discussions. Deep learning approaches have attempted to address these limitations with varying degrees of success. Xie et al. [24] pioneered social media AE detection using Bi-LSTM architecture for e-cigarette-related events, while Bergman et al. [27] developed AER-BERT specifically for Swedish adverse drug reaction reports. However, as shown in **Table 2**, these approaches have been constrained by narrow focus areas, language limitations, or limited integration with diverse social media platforms. Our study extends this growing body of literature by using a routinized approach that can be adopted broadly to efficiently and effectively identify AEs from unstructured text data.

**Table 2:** Comparison of Waldo with existing AE detection tools.

Tool	Model Architecture	Data Source & Domain Focus	Performance Metrics	Availability	Key Limitations
**Waldo**	RoBERTa fine-tuned	Reddit posts - Cannabis-derived products	Accuracy: 99.7%, F1: 0.95, Precision: 93.6%, Recall: 96.4%	Open-source	Limited to cannabis products; requires human review
**adverse-events [26]**	Traditional ML (SVM, Random Forest)	FAERS database - General pharmaceuticals	Variable (dataset-dependent)	Open-source	Narrow focus on structured FAERS data; limited NLP capabilities
**AER-BERT [27]**	BERT-based classification	ADR reports from Sweden - General pharmaceuticals	Accuracy: 82.7%, F1: 0.72, Precision: 79.6%, Recall: 65.8%	Academic tool	Swedish-language ADR reports; limited social media integration
**E-cigarette AE Detection [24]**	Bi-LSTM with embeddings	E-Cigarette forum posts - E-cigarettes	F1: 0.93, Precision: 94.1%, Recall: 91.8%	Research code only	Single platform; narrow product focus
**WebMD-AE-Extractor [25]**	Support Vector Machine	WebMD patient reviews - Diabetes medications	Precision: 0.69, Recall: 0.71	Research prototype	Limited to specific therapeutic area; traditional ML approach

Waldo represents a significant advancement in social media-based AE detection, achieving superior performance compared to existing approaches. Waldo’s superiority stems from combining state-of-the-art RoBERTa architecture with comprehensive social media integration, specifically targeting the underserved cannabis market. Unlike previous tools limited to specific platforms [24], languages [27], or narrow therapeutic areas [25], Waldo addresses cannabis-derived products—a rapidly growing segment with regulatory gaps in post-market surveillance.

Additionally, Waldo’s automated approach has broad applicability beyond cannabis-derived products to other consumer health products that similarly lack regulatory oversight. The methodology could be readily adapted to monitor dietary supplements and vaping products, where machine learning approaches have successfully detected adverse events from social media platforms [24,28]. This versatility is particularly valuable given that many consumer health products fall into regulatory gaps—dietary supplements operate under the Dietary Supplement Health and Education Act (DSHEA) with limited pre-market safety requirements, while e-cigarettes face evolving frameworks that lag behind market penetration. By providing an automated, cost-effective tool for AE detection from consumer narratives, Waldo-like systems could fill surveillance gaps across multiple product categories and identify safety signals that might otherwise go undetected until reaching clinical significance.

As we turn our attention to cannabis-derived products, a category that has largely evaded federal oversight in marketing [29], we focus on addressing the unique safety challenges they present through enhanced monitoring efforts. Waldo can play a crucial role in making safety monitoring more commonplace for CDPs, especially given their rising use among individuals with multiple chronic conditions [30]. Recent studies have highlighted drug-drug interactions between immunosuppressants and CDPs, including THC and CBD [31,32], emphasizing the need for vigilance. By applying Waldo to analyze posts from 20 different subreddits, we identified certain communities, such as r/weed, showing particularly high AE rates.

The findings from this study have several important implications. First, they demonstrate that unstructured text data can be a valuable resource for pharmacovigilance. Waldo’s ability to detect AEs from various subreddits not only broadens the scope for AE detection but also democratizes access to safety surveillance, moving beyond the industry-centric models that have traditionally dominated. This means that underserved topics which have historically been neglected – like CDPs – will benefit. By identifying subreddits with high AE rates, researchers and public health officials can target their efforts more precisely, thereby reducing the proverbial haystack and increasing the likelihood of finding relevant needles.

Beyond research applications, Waldo can support clinicians by surfacing real-world patient experiences with CDPs that may otherwise go unreported. For instance, clinicians could review Waldo-flagged AEs to better counsel patients on potential risks or identify patterns (e.g., panic attacks with high-THC products) relevant to individual care. Integrating Waldo into clinical dashboards, EMR alerts, or patient-facing education tools could help bridge the current gap between informal health narratives and formal clinical decision-making.

Waldo’s open-source nature further democratizes access to advanced AE detection technology, encouraging further development within the research community. Typically, researchers mine social media for all drug mentions and then search these for any safety signals, but this approach has significant drawbacks [1]. Moreover, Waldo has outperformed an LLM-powered chatbot for AE detection. While chatbots in healthcare offer automation potential, they broadly struggle with issues of reproducibility, transparency, and cost-effectiveness [33]. Chatbot models evolve, they may not perform consistently on new data sets, and their decision-making features are unclear [33]. Furthermore, their dependency on precise user inputs and prompting can lead to errors in critical tasks like AE detection. In contrast, Waldo is specifically designed for AE detection, offering higher accuracy and reliability. Its availability on GitHub makes it a cost-effective alternative that doesn’t compromise on performance, ultimately enhancing patient safety and elevating the quality of medical research outcomes.

However, there are also limitations that need to be addressed. Currently, Waldo relies on human investigators to review posts, which introduces a bottleneck in the process. Future iterations could implement confidence-based prioritization using predicted probabilities from the model to automatically classify posts with very high or low confidence scores while flagging intermediate cases for human review. Additionally, incorporating an active learning framework where uncertain predictions are iteratively labeled by human reviewers and fed back into model retraining could progressively reduce the human review burden over time [34]. While these technical improvements may increase efficiency, they must be balanced against the critical need for human oversight. Over-reliance on automated outputs without adequate human validation poses significant risks, as false positives could create unnecessary safety alarms while false negatives could miss critical signals. Therefore, healthcare providers and regulatory agencies must maintain human oversight, using Waldo as a screening tool rather than definitive diagnostic instrument. Organizations implementing Waldo should establish clear human review protocols and ensure transparency about automated screening processes in pharmacovigilance applications.

While Waldo was trained on posts from r/Delta8, this delta-8-THC-specific dataset may introduce bias, potentially limiting generalizability to other cannabis-derived products or user communities. Future work should evaluate Waldo’s performance on a broader range of product types and forums to assess robustness across different AE types. Moreover, our comparison with ChatGPT utilized default parameter settings without optimization, which may not reflect the model’s full potential under tuned conditions, though the substantial performance differences observed suggest that parameter optimization would be unlikely to close the performance gap for this specialized task. Future model refinement through additional training data and more sophisticated NLP techniques will be crucial. Collaborations with regulatory bodies could facilitate the integration of Waldo into existing safety surveillance systems, making AE reporting more efficient.

In conclusion, this study demonstrates the significant potential of automated machine learning tools like Waldo to enhance AE detection by harnessing unstructured text data from social media. By democratizing access to safety surveillance and improving the efficiency of AE reporting, tools like Waldo represent a promising step forward in the evolution of pharmacovigilance.

## Supporting information

S1 File Supplementary Material. (DOCX)
