# Supplementary material for: Waldo: Automated discovery of adverse events from unstructured self reports
Source: PLOS Digit Health. 2025 Sep 30;4(9):e0001011. doi: 10.1371/journal.pdig.0001011 (PMC12483200; doi:10.1371/journal.pdig.0001011)
Supplement: S1 File — (DOCX) [file pdig.0001011.s001.docx]

**Supplement A**

In the results we reported the accuracy for the pooled analysis comparing all 10,000 true labels against those from Classifier C. Here we report the results disaggregated for the test period alone, where the training set comprised 90% of the data and the test set 10% of the data.

Classifier A (N-gram Model) correctly predicted 97.1% of outcomes, with 0 false positives and 29 false negatives (F1=0.22 for the positive class) **(Fig A)**. Classifier B (BERT), with an accuracy of 97.9%, had 9 false positives and 12 false negatives (F1=0.67 for the positive class) (Fig 1). Classifier C (RoBERTa) delivered the highest accuracy at 97.9%, with 11 false positives and 10 false negatives (F1=0.69 for the positive class**)**. Furthermore, Classifier C had a precision of 67.7% (the proportion of true positive predictions among all positive predictions) and a recall of 69.7% (the proportion of true positive predictions among all actual positive instances). Regardless of using the pooled analysis or the test analysis, the same method (Model C: RoBERTa) would be selected.

**Supplement B**

The AI-powered chatbot was assessed on the full dataset of 10,000 samples to evaluate its capability in detecting adverse events (AEs) compared to the human annotator model, Waldo. The chatbot achieved an overall accuracy of 94.4%, correctly classifying 9,436 out of 10,000 instances **(Fig B)**. However, it exhibited a higher misclassification rate, with 401 false positives and 163 false negatives—representing 18.2 times more false positives and 13.6 times more false negatives than Waldo.

The chatbot's precision was 30.0%, indicating that only 30% of its positive predictions were correct. Its recall was 51.3%, successfully identifying just over half of the actual AEs present in the dataset. The F1 score for the positive class was 0.38, substantially lower than Waldo's F1 score of 0.95. These metrics highlight a significant underperformance in the chatbot's ability to detect AEs compared to the specialized model accurately.

These findings suggest that while the AI-powered chatbot demonstrates moderate overall accuracy, its effectiveness in AE detection is limited due to high rates of false positives and false negatives. The results underscore the importance of specialized models like Waldo for precise AE identification in unstructured text data.

**
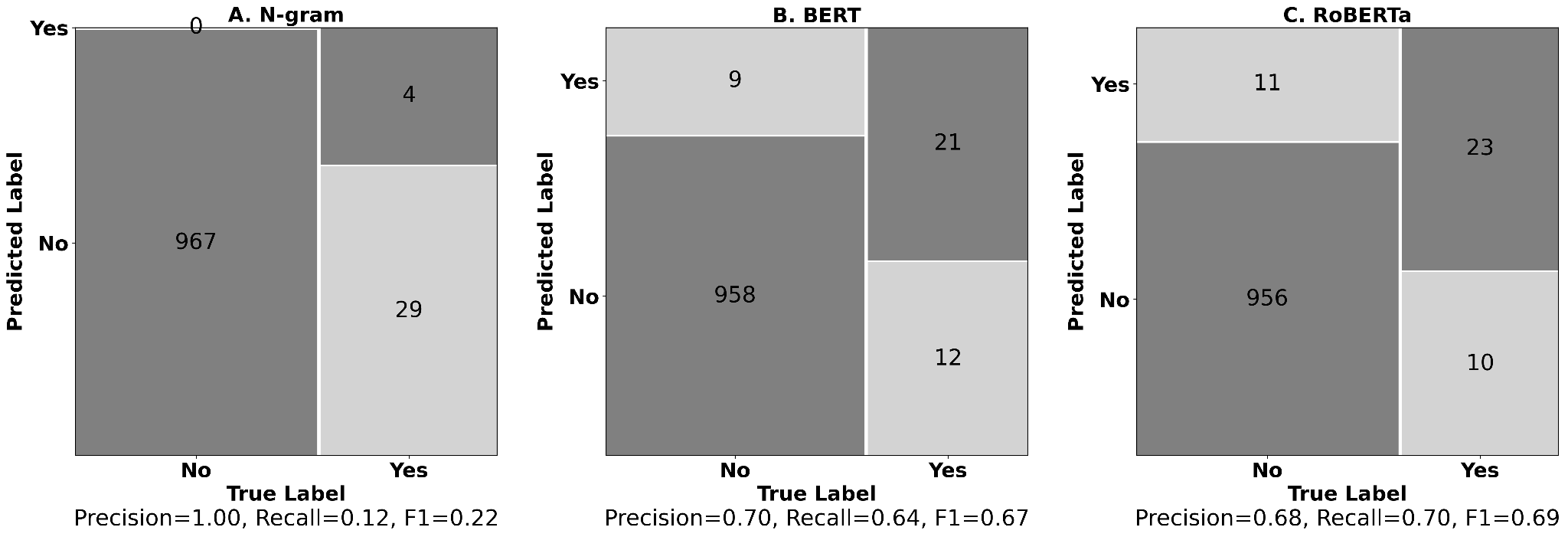
**

**Fig A: Confusion Matrices for Three Models on Test Analysis**

Comparison of confusion matrices for N-gram (A), BERT (B), and RoBERTa (C) classifiers.


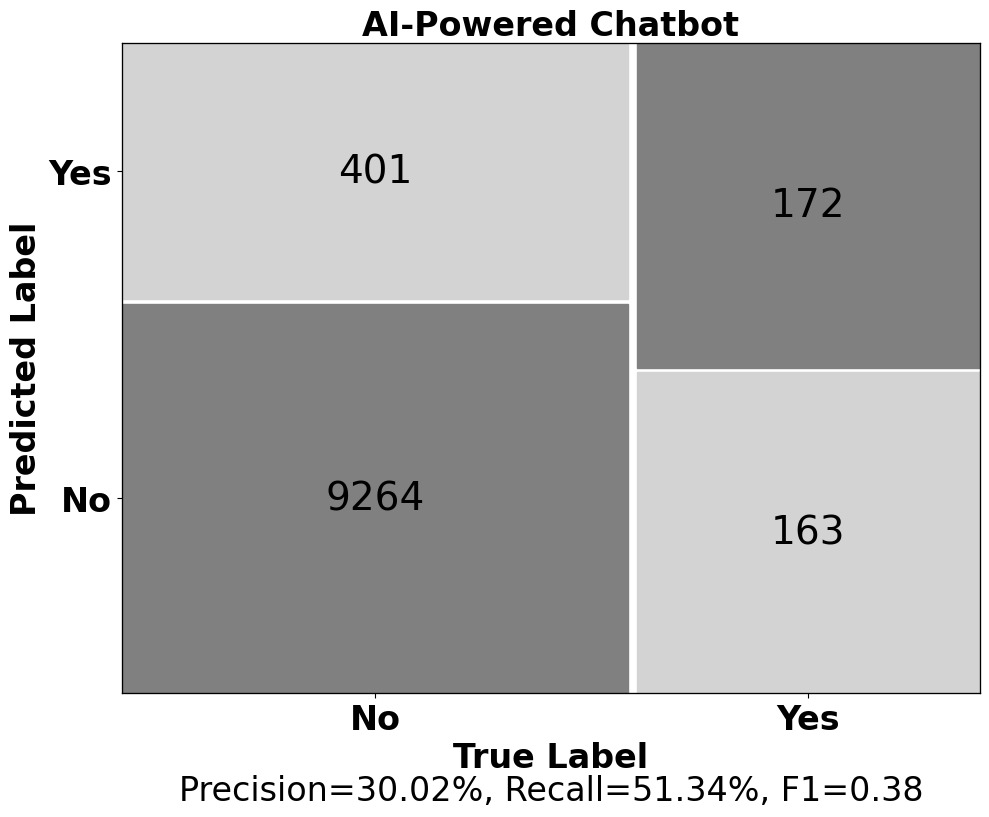


**Fig B: Confusion Matrix for AI-Powered Chatbot**
